# The effect of fluoroquinolones and antioxidans on biofilm formation by *Proteus mirabilis* strains

**DOI:** 10.1186/s12941-022-00515-5

**Published:** 2022-06-02

**Authors:** Jana Przekwas, Jakub Gębalski, Joanna Kwiecińska-Piróg, Natalia Wiktorczyk-Kapischke, Ewa Wałecka-Zacharska, Eugenia Gospodarek-Komkowska, Dorota Rutkowska, Krzysztof Skowron

**Affiliations:** 1 Department of Microbiology, Nicolaus Copernicus University in Toruń, L. Rydygier Collegium Medicum in Bydgoszcz, Bydgoszcz, Poland; 2 Department of Pharmaceutical Botany and Pharmacognosy, Nicolaus Copernicus University in Toruń, L. Rydygier Collegium Medicum in Bydgoszcz, Bydgoszcz, Poland; 3 Department of Food Hygiene and Consumer Health, Wrocław University of Environmental and Life Sciences, Wrocław, Poland; 4 Medical Education Unit, Nicolaus Copernicus University in Toruń, L. Rydygier Collegium Medicum in Bydgoszcz, Bydgoszcz, Poland

**Keywords:** Biofilm, *Proteus mirabilis*, Antioxidants, Antibiotics

## Abstract

**Background:**

Fluoroquinolones are a group of antibiotics used in urinary tract infections. Unfortunately, resistance to this group of drugs is currently growing. The combined action of fluoroquinolones and other antibacterial and anti-biofilm substances may extend the use of this therapeutic option by clinicians. The aim of the study was to determine the effect of selected fluoroquinolones and therapeutic concentrations of ascorbic acid and rutoside on biofilm formation by *Proteus mirabilis*.

**Materials and methods:**

The study included 15 strains of *P. mirabilis* isolated from urinary tract infections in patients of the University Hospital No. 1 dr A. Jurasz in Bydgoszcz (Poland). The metabolic activity of the biofilm treated with 0.4 mg/ml ascorbic acid, 0.02 µg/ml rutoside and chemotherapeutic agents (ciprofloxacin, norfloxacin) in the concentration range of 0.125–4.0 MIC (minimum inhibitory concentration) was assessed spectrophotometrically.

**Results:**

Both ciprofloxacin and norfloxacin inhibited biofilm formation by the tested strains. The biofilm reduction rate was correlated with the increasing concentration of antibiotic used. No synergism in fluoroquinolones with ascorbic acid, rutoside or both was found. The ascorbic acid and rutoside combination, however, significantly decreased biofilm production.

**Conclusions:**

Our research proves a beneficial impact of ascorbic acid with rutoside supplementation on biofilm of *P. mirabilis* strains causing urinary tract infections.

## Introduction

Biofilm is a community of microorganisms encased in a self-produced matrix. The matrix consists of exopolysaccharides, proteins, and nucleic acids and participates in the adhesion of bacteria to the surface. It is also a barrier protecting bacteria against adverse environmental conditions, e.g., drying and phagocytosis. Bacterial cells in a biofilm are many times more resistant to antibiotics than those in the planktonic form. On the surface of the polysaccharide capsules, there are free functional groups capable of adsorbing antibiotics [1, 2]. The expression of some genes encoding pump proteins is activated exclusively in the biofilm environment. Protein pumps contribute to resistance to tetracyclines, fluoroquinolones, macrolides, and beta-lactams [2, 3]. Growth conditions play an important role in biofilm resistance. Cells located in deeper layers of the biofilm have limited access to nutrients, but their slow metabolism reduces their sensitivity to antibiotics [4, 5]. Insufficient oxygen supply leads to changes in the metabolism of bacteria. Then, they carry out incomplete glucose oxidation, resulting in the formation of acidifying compounds. Changes in pH lead to the inactivation or ionization of the antibiotics and impede penetration through the cell wall or cell membrane [3, 6].

Biofilm is a structure formed by all microorganisms on different types of surfaces. Of great importance are biomaterials used in the patient's body, e.g., urinary bladder catheters [7]. Numerous bacteria, including uropathogens, take part in their colonization. Among them, the Gram-negative rod of the species *Proteus mirabilis* is of particular concern. The bacterium produces urease leading to struvite (ammonium phosphate) and apatite (calcium phosphate) deposits in the catheter lumen [8–10]. Inability to drain urine increases the risk of infection, creating an excellent environment for bacteria to grow. This can contribute to further consequences such as pyelonephritis and bacteremia [11].

Fluoroquinolones are a group of antibiotics used in urinary tract infections (UTI). They are active against Gram-negative rods that most commonly cause UTIs. Fluoroquinolones prevent replication by inhibiting bacterial topoisomerase type II (gyrase) and type IV action [12]. This leads to DNA damage and reactive oxygen species (ROS) production responsible for oxidative stress [13].

Treating biofilm-associated infections is difficult. Therefore, it seems reasonable to research materials that will inhibit biofilm formation, preventing the massive dispersion of microbial cells in the human body. It is also essential to look for synergies between well-known compounds, e.g., antibiotics and vitamins or antibiotics and polyphenols. Ascorbic acid (vitamin C) functions mainly as a cofactor of the enzyme involved in the hydroxylation during collagen biosynthesis [14]. Due to its strong reducing properties, it is the most important antioxidant active against ROS released by phagocytes. Ascorbic acid has antibacterial properties by inducing oxidative stress in bacterial cells [15, 16]. It also hinders biofilm development, among others: *Mycobacterium* spp., *Staphylococcus aureus*, *Escherichia coli*, *Listeria monocytogenes,* or *Pseudomonas aeruginosa, by* inhibiting the production of exopolysaccharides [15, 17–19]. Rutoside is a compound from the group of polyphenols. It is an antioxidant with antifungal, antiviral, and antibacterial properties. The antimicrobial effect is due to interaction with the cell wall. The resulting complexes disrupt the integrity, block ion channels and inhibit the electron transport responsible for the synthesis of ATP [20, 21]. Rutoside also inhibits the action of bacterial type IV topoisomerase and induces an SOS response by inducing oxidative stress in *E. coli* [22]. It has also anti-biofilm activity. It inhibits the secretion of type II autoinductors and the operation of efflux pumps [23, 24]. Rutoside, in addition to its antibacterial effect, prolongs the action of ascorbic acid by stabilizing it and forming complexes with copper ions, preventing the vitamin C oxidation reaction, which is crucial during biofilm eradication [25].

This study aimed to determine the effect of fluoroquinolones (norfloxacin and ciprofloxacin) and antioxidants (ascorbic acid and rutoside) on biofilm formation by *Proteus mirabilis* strains isolated from catheterized patients. Both, fluoroquinolones and mentioned above antioxidans, are common prescribed in UTIs treatment. Since, for example, rutoside and fluorochinolones takes the same place of action (topoisomerase IV), these molecules may cause antagonistic effect.

## Materials and methods

### Tested strains

The study was conducted on 15 *P. mirabilis* strains isolated from urinary tract infections in patients of the University Hospital No. 1 dr A. Jurasz in Bydgoszcz (Poland). Strains were included based on the antimicrobial susceptibility testing results (Phoenix M50, N-302 panels) in order to obtain two groups of strains: resistant and susceptible to examined fluoroquinolones. Species identification was performed using mass spectrometry (Microflex, Bruker). The strains were stored at − 70 °C in Brain Heart Infusion (BHI; Becton Dickinson) enriched with 10.0% glycerol (Avantor). Before analysis strains were plated on blood agar (Columbia Agar; Becton Dickinson) and incubated at 37 °C for 24 h. All tested strains were proven to produce biofilm by crystal violet assay.

### Test compounds

#### Antibiotics

Two antibiotics were used i.e., ciprofloxacin and norfloxacin (Sigma Aldrich). The fluoroquinolones were dissolved according to CLSI recommendations in a Phosphate Buffered Saline (PBS; Becton Dickinson) at pH 7.2. In order to increase the solubility of fluoroquinolones, 1 M NaOH (Avantor) solution was used.

The minimum inhibitory concentration (MIC) was assessed using micro-dilution method in a liquid medium in accordance with the CLSI recommendations [26].

The impact of examined antibiotics in range between 0.25 – 4 MIC value on biofilm formation by *P. mirabilis* strains was studied.

#### Ascorbic acid

Ascorbic acid was dissolved in Mueller–Hinton broth (MHB; Becton Dickinson) to give a final concentration of 0.4 mg/ml.

#### Rutoside

Rutoside was dissolved in Mueller–Hinton broth (MHB; Becton Dickinson) to a final concentration of 0.02 µg/ml.

### Assessment of the influence of fluoroquinolones on biofilm activity

**Fig. 1:**
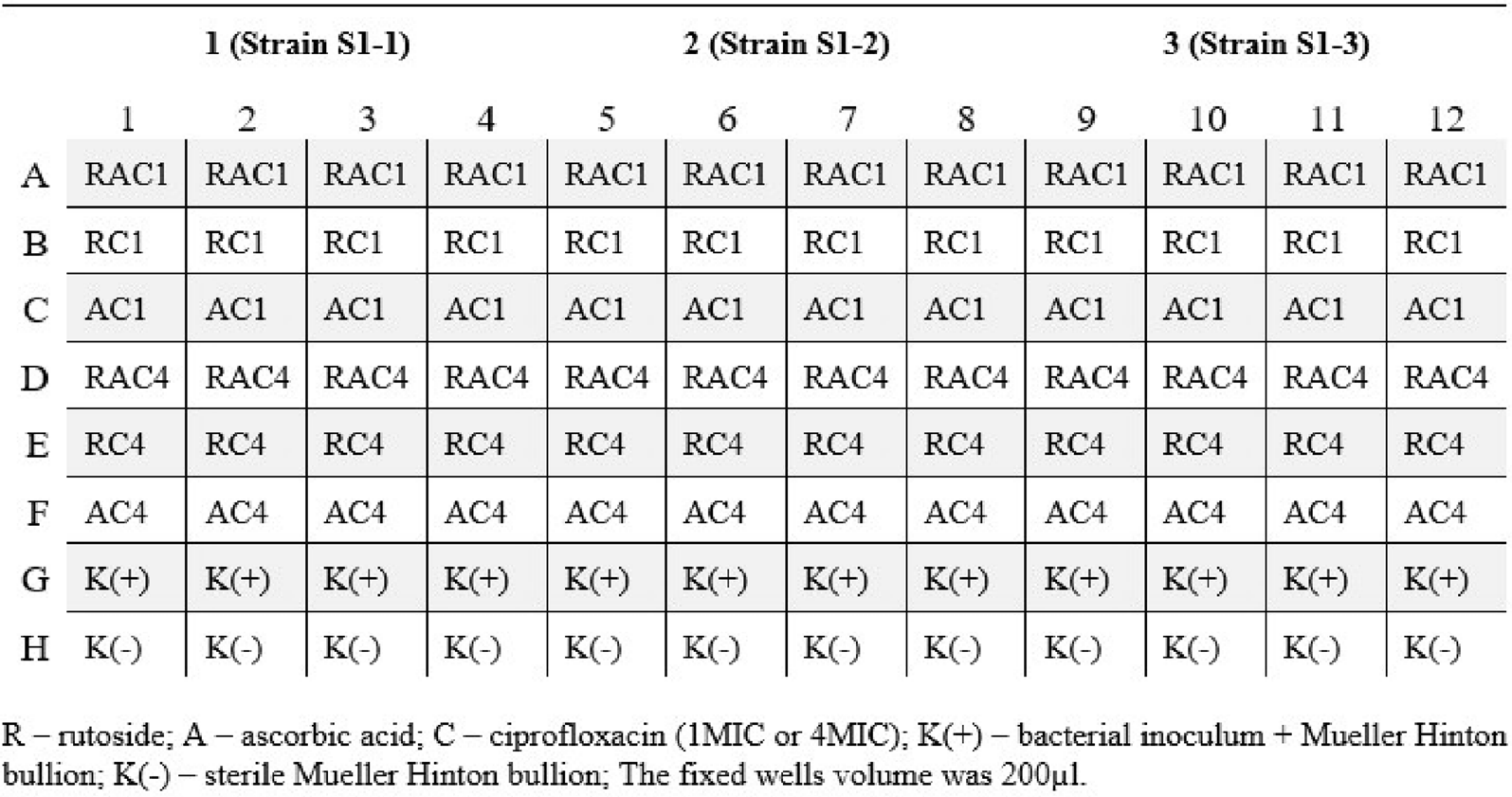
Plan of arrangement on 96-wells plate

The influence of the tested compounds on biofilm formation was evaluated using 96-well titration plates (Nest Biotechnology Co., Ltd.). For each strain bacterial suspension of approx. 5 × 10^5^ CFU/ml (100 µl) was treated with 100 µl of chemotherapeutic concentration of 1MIC, two higher concentrations (4MIC and 2MIC) and three sub-inhibitory concentrations (0.5MIC, 0.25MIC, and 0.125MIC). Plates were incubated at 37 °C for 24 h. Next, the plates were rinsed twice with PBS and each well replenished with 180 µl of trypticase-soy bullion (TSB; Becton Dickinson) and 20 µl 2, 3, 5-Triphenyl-tetrazolium chloride (TTC; Avantor). The prepared plates were incubated at 37 °C for 4 h. After incubation, the dye was rinsed with water until colorless washings. Then, 300 µl of methanol (Avantor) was added to the dried wells (Fig. 1). Biofilm formation was determined by measuring formazan absorbance at 470 nm (Synergy, BioTek) and archived in the KC4 program (BioTek).

### Study of the influence of fluoroquinolones, ascorbic acid and rutoside on biofilm formation

Two concentrations of antibiotics, 1MIC and 4MIC, in combination with rutoside and ascorbic acid, rutoside only and no antioxidants were tested (Fig. 1). The remaining activities were carried out as described in the previous section.

### Analysis of the results

The obtained results were saved in KC4 (BioTek) and then processed in Microsoft Office Excel (2007). The statistical analysis was performed using the Statistica 13.1 PL program [TIBCO Software Inc. (2017). Statistica (data analysis software system], version 13. http://statistica.io). Values were considered statistically significant at p < 0.05.

The influence of tested compounds on biofilm formation was analyzed using dependent variable comparison tests. To determine the differences between strains susceptible and resistant to fluoroquinolones, the coefficient of biofilm reduction for the tested compound was calculated. Then the results were compared using tests for independent variables in the groups. The comparison of absorbance values between strains was impossible due to the strain-specific metabolism of TTC to formazan.

The formula for the biofilm reduction ratio:

$$ bio{\mkern 1mu} film{\mkern 1mu} \,reduction\,{\mkern 1mu} ratio\left[ \%  \right] = \frac{{the\,{\mkern 1mu} absorbance\,{\mkern 1mu} value\,{\mkern 1mu} of{\mkern 1mu} the{\mkern 1mu} positive\,{\mkern 1mu} control - the{\mkern 1mu} \,value\,{\mkern 1mu} of{\mkern 1mu} the{\mkern 1mu} \,absorbance\,{\mkern 1mu} of{\mkern 1mu} the{\mkern 1mu} \,test{\mkern 1mu} sample}}{{the{\mkern 1mu} \,absorbance\,{\mkern 1mu} value{\mkern 1mu} \,of{\mkern 1mu} the{\mkern 1mu} positive{\mkern 1mu} \,control}} \times 100\%  $$

The positive values of the reduction coefficient indicated a reduction of the biofilm under the influence of the tested compound, while the negative values an increase in the metabolic activity of the biofilm.

## Results

### MIC values of fluoroquinolones for *P. mirabilis*

MIC values were interpreted according to the EUCAST (European Committee on Antimicrobial Susceptibility Testing) recommendation [27]. Strains were classified based on the MIC value as: susceptible (S) or resistant (R) (Table 1). Amongst examined 15 strains, 7 (46.7%) were found as susceptible to both fluoroquinolones, and 8 (53.3%) – as resistant.

**Table 1:** MIC values of ciprofloxacin and norfloxacin and interpretation

Strain	MIC			
	Ciprofloxacin		Norfloxacin	
	Value [μg/ml]	Interpretation	Value [μg/ml]	Interpretation
S1 (2270)	1	R	4	R
S3 (2166)	2	R	4	R
S9 (2309)	0.016	S	0.063	S
S11 (2037)	0.016	S	0.125	S
S12 (2119)	0.063	S	0.25	S
S13 (2114)	2	R	8	R
S14 (2285)	2	R	8	R
S15 (2314)	0.016	S	0.125	S
S16 (2348)	0.016	S	0.063	S
S17 (2249)	0.125	S	0.125	S
S18 (2266)	0.032	S	0.125	S
S19 (2291)	4	R	16	R
S20 (2140)	32	R	64	R
S21 (2246)	1	R	4	R
S22 (2274)	2	R	4	R

### Impact of fluoroquinolones on biofilm formation

**Fig. 2:**
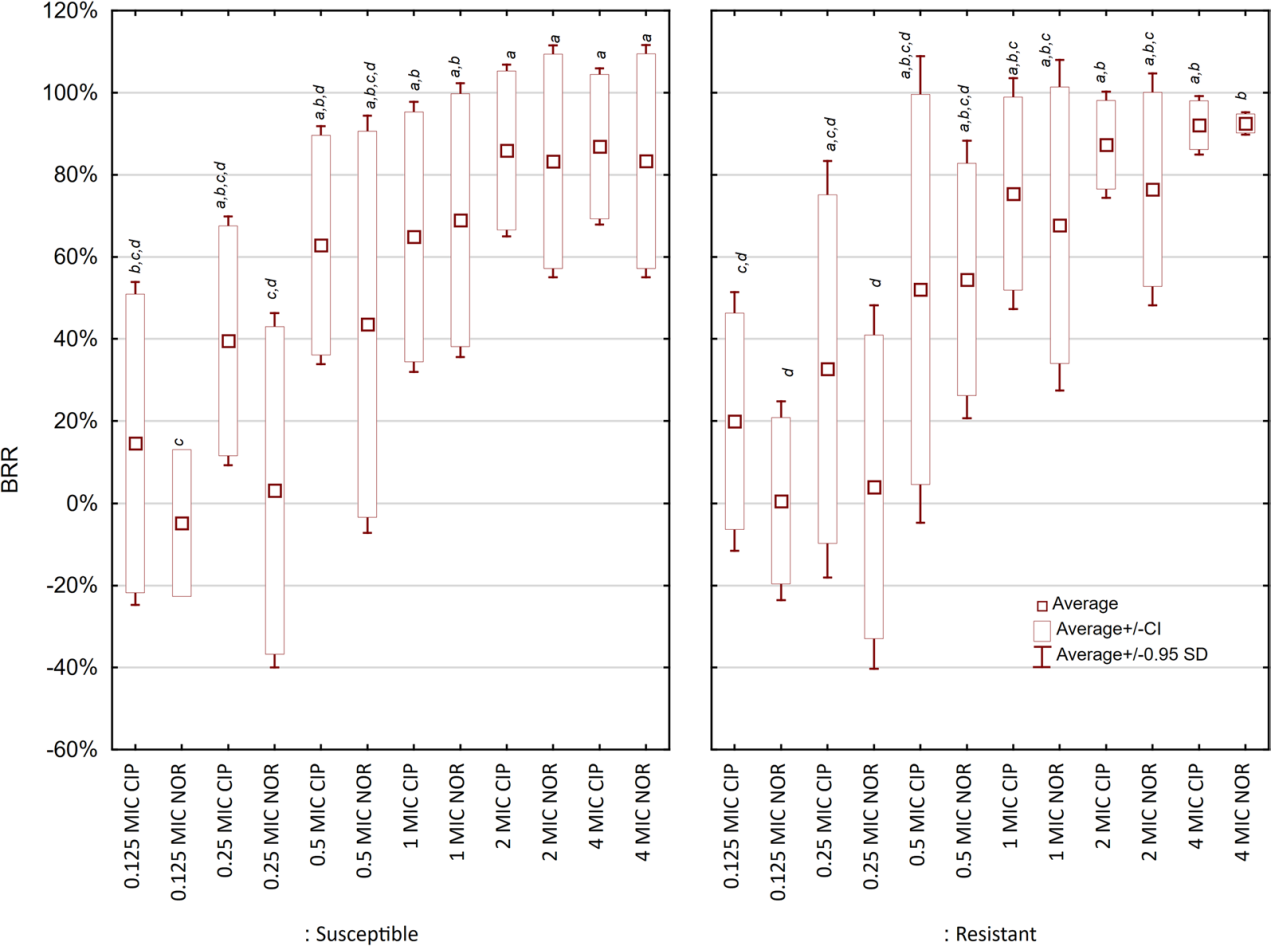
Impact of fluroquinolones on biofilm formation by *P. mirabilis* strains (n = 15)

In the first step of investigation, we examined the impact of 6 different concentration of ciprofloxacin and norfloxacin (0.125MIC, 0.25 MIC, 0.5 MIC, 1MIC, 2MIC, 4MIC) on biofilm formation by fluoroquinolones-resistant (FR) and – susceptible (FS) *P. mirabilis* strains. The highest biofilm reducing ration (BRR) values were observed in the concentration corresponding to MIC = 4, achieving the 80–90% (Fig. 2). The inhibition effect of ciprofloxacin on biofilm formation was observed even in the lowest examined concentrations (0.125 of MIC), but it was insignificant. The lowest examined concentration of norfloxacin (MIC 0.125) promoted biofilm formation in the group of FS strains by approx. 5% (not significant).

We found that BRR increased together with the antibiotic concentration. Ciprofloxacin shows slightly stronger impact on biofilm reduction than norfloxacin in concentrations below 1MIC, but the difference is not statistically significant. In concentrations above 1MIC there is no difference between both antibiotics for susceptible and resistant strains.

### Impact of antioxidants on biofilm formation

**Fig. 3:**
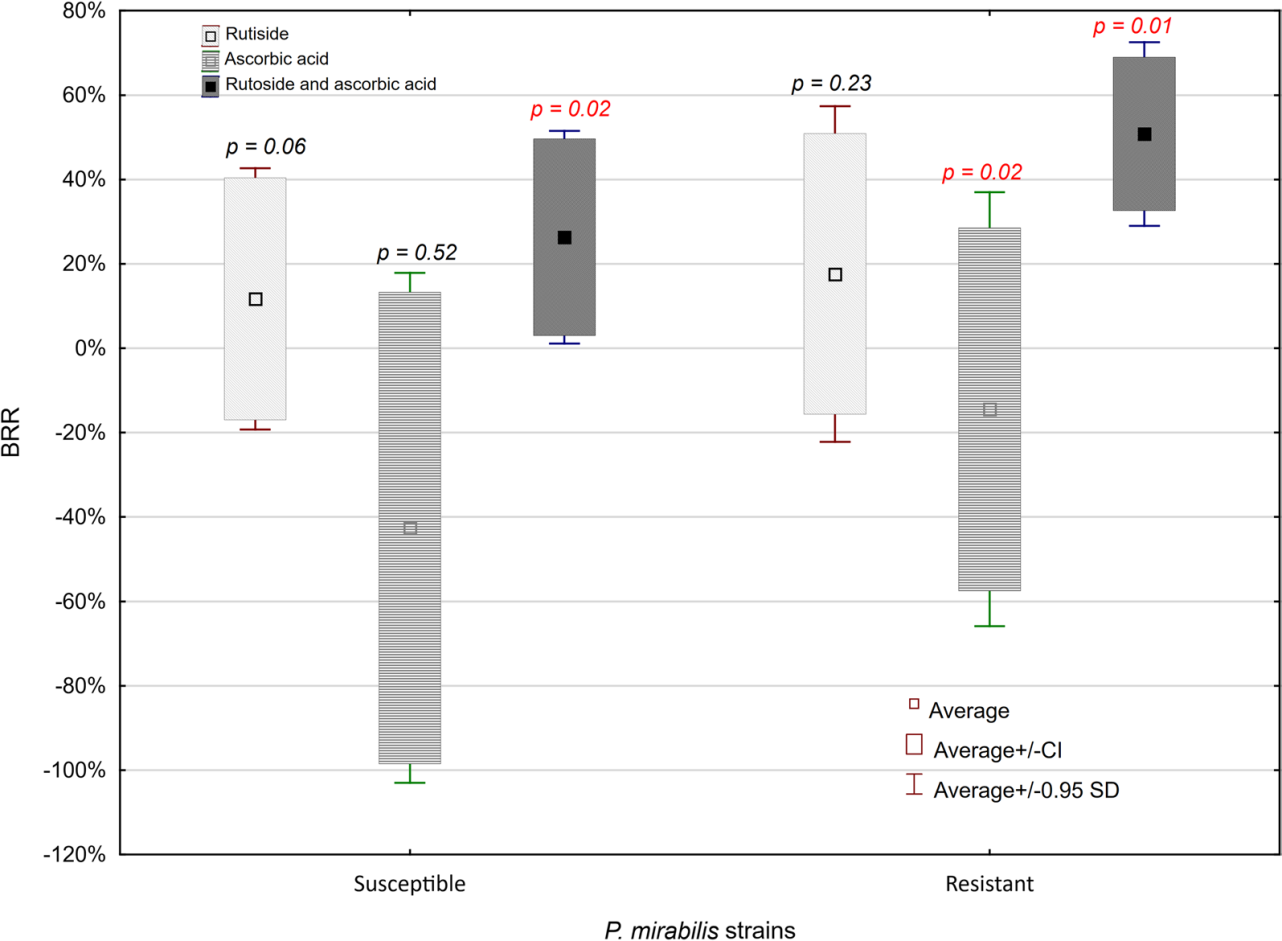
Impact of antioxidants on biofilm formation by *P. mirabilis* strains (n = 15)

We examined the impact of 0.02 µg/ml rutoside or/and 0.4 mg/ml of ascorbic acid on biofilm formation of the 15 *P. mirabilis* strains. We found that 0.4 mg/ml of ascorbic acid promoted biofilm formation. The BRR average was under 0% both in FS and FR strains groups, and it was statistically significant in FR group (Fig. 3). The treatment of rutoside and rutoside with ascorbic acid caused the inhibition of biofilm formation in both groups. The BRR values for rutoside were 11.7% in FS and 17.6% in FR group (not sigificant). For rutoside with ascorbic acid BBR values were 26.3% for FS and 50.8% for FR group (p < 0.05). The BRR value obtained for rutoside and ascorbic acid in FR strains group was similar to BRR values obtained in the presence of 0.5 MIC fluoroquinolones (ciprofloxacin 52.1%; norfloxacin 54.5%) (Fig. 3).

### Impact of combined effect of fluoroquinolones and antioxidants on biofilm formation

**Fig. 4:**
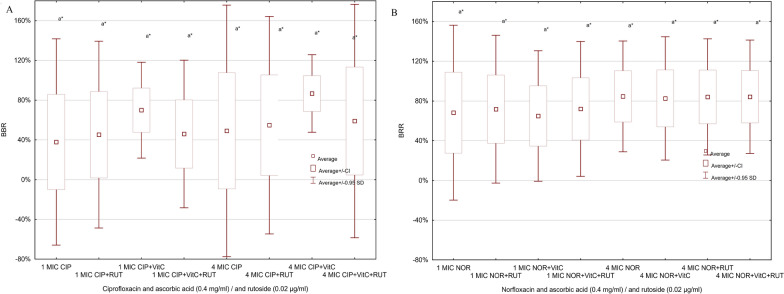
Impact of fluoroquinolones (4**A**—ciprofloxacin, 4**B**—norfloxacin) and antioxidants on biofilm formation by *P. mirabilis* fluoroquinolones susceptible strains (n = 7)

We examined the combined impact of fluoroquinolones (two concentrations: 1 MIC and 4 MIC), rutoside (0.02 µg/ml) and/or ascorbic acid (0.4 mg/ml) on biofilm formation by FS and FR strains of *P. mirabilis*. Amongst FS strains, the highest BRR value (86.7%) was observed in the presence of ascorbic acid and 4 MIC of ciprofloxacin (Fig. 4a). It was higher than 4 MIC CIP alone (49.1%) and other combinations (not significant). Similar relationship was found in 1 MIC concentration of ciprofloxacin and combinations, the highest BRR value (68.5%) was obtained for 1 MIC ciprofloxacin and ascorbic acid (Fig. 4a) (not significant). For norfloxacin BRR average values were very similar in every examined combination (Fig. 4b).

**Fig. 5:**
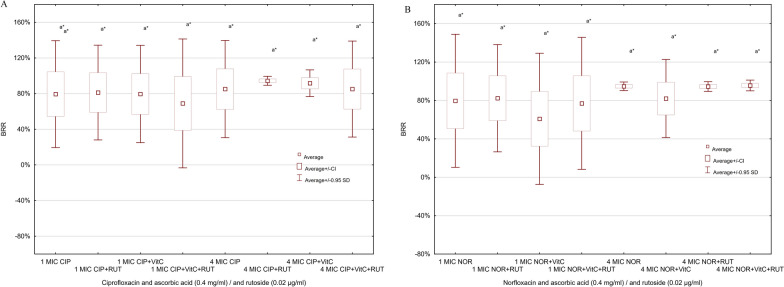
Impact of fluoroquinolones (5**A**—ciprofloxacin, 5**B**—norfloxacin) and antioxidants on biofilm formation by P. mirabilis fluoroquinolones-resistant strains (n = 8)

In FR strains group the BRR average values range between 80–90% (Fig. 5a and b). The highest differences between BRR values were observed for combination of 4 MIC of ciprofloxacin and examined antioxidants (85.1%). The lowest BRR values were obtained for 1MIC norfloxacin with ascorbic acid (60.9%). The difference between 1MIC antibiotics and 4MIC antibiotics is visible, but it is not statistically significant (p > 0.05).

## Discussion

*Proteus* rods account for 1 to 2% of all UTIs, while in complicated UTIs have a higher frequency—from 20 to 45% [29]. Susceptibility of *P. mirabilis* strains to many antimicrobials, including ciprofloxacin decreased significantly during the past decade, from 80.1 to 53.8% [30].

Therefore, the use of fluoroquinolones in empiric therapy requires particular caution. One solution is the combination of antibiotics and other compounds with antibacterial or anti-biofilm properties.

Based on the obtained results, both tested fluoroquinolones show anti-biofilm activity. Each of the tested antibiotics in concentrations of 2 and 4 MIC reduced the biofilm by approx. 80–90%. Considering that 4MIC is easily obtainable in urine during antibiotic therapy against UTI (for susceptible strains), fluoroquinolones may have therapeutic effect against biofilm formed in urinary tract. Researchers confirm the anti-biofilm activity of fluoroquinolones against Gram-negative rods [31–36]. However, what limits the action of the antibiotic in vivo is its bioavailability. Due to a wide spectrum of action, fluoroquinolones are used in empirical therapy.

Ciprofloxacin is the best choice, among fluoroquinolones, against Gram-negative rods infections. It has the highest pharmacodynamic indexes compared to other antibiotics [37] and is effective for both oral and intravenous administration [38]. The concentration of fluoroquinolones in the urine is 100 or even 1000 times higher than in the serum. These drugs effectively eradicate the bacterial biofilm and are a therapeutic option for UTIs [39]. Researchers have concluded that fluoroquinolones (mainly cipro—and levofloxacin) may be effective in mild cases of UTIs caused by Enterobacterales strains with MIC ≤ 32 in vitro*.* However, they must be administered at high doses to avoid the selection of resistant strains [11]. The latest guidelines recommend the use of fluoroquinolones (cipro- or levofloxacin) as the first-line drug in uncomplicated nephritis and cystitis accompanied by kidney stones (urolithiasis), commonly caused by *P. mirabilis* [11, 40, 41]. In other cases (uncomplicated UTI, complicated UTI, catheter associated UTI) they are second-line drugs. They are not recommended if the resistance of *Enterobacteriaceae* to this group of drugs is higher than 10% [40]. Norfloxacin, used for years in upper and lower UTI, is not currently listed as a therapeutic option in the latest recommendations of the European Urological Association [40].

In our study we have checked the interaction of antioxidants on biofilm formation by *P. mirabilis* strains. The one antioxidant used in this study was rutoside. It reduced the biofilm only from 11.7 to 17.6%, in fluoroquinolones-susceptible, and – resistant strains, respectively. Numerous studies examining the impact of polyphenolic compounds solutions or plant extracts confirm the antibacterial and anti-biofilm properties. This activity is likely associated with the ability to form complexes with the cell wall or proteins contained in the plasma membrane or cytoplasm [41–50]. Many research groups have also shown synergistic antibacterial activity of two or three polyphenolic compounds (quercetin, morin, rutoside) against Gram-positive and Gram-negative bacteria [51–54]. However, based on our research, rutoside may not reduce biofilm effectively alone.

The obtained results show that rutoside and vitamin C do not significant affect biofilm formation, but their combination statistically significantly inhibits biofilm formation. The available literature does not explain the observed phenomenon. This effect may be related to the pro-oxidative properties of ascorbic acid, revealed under specific conditions such as low concentration, the presence of transition group metals (iron), and stabilization in the presence of polyphenol (rutoside) [55–60]. However, the issue requires further research. Such research should include the determination of free radical reaction markers' presence, e.g., malondialdehyde, in the reaction medium, using analytical methods (high performance liquid chromatography—HPLC).

One objective of the study was to determine the effect of ascorbic acid on the anti-biofilm activity of fluoroquinolones. The applied concentration corresponds to these obtained in the urine after supplementation with the maximum recommended dose. Ascorbic acid at the concentration reached in urine promoted biofilm formation by *P. mirabilis* strains. We have found no statistically important synergy between fluoroquinolones and vitamin C at the therapeutic concentration (0.4 mg/ml). Goswami et al. [61] and Masahed et al. [62] using, i.e. ascorbic acid decreased the effectiveness of fluoroquinolones against *E. coli* strains. On the contrary, El-Gebaly et al. [63] have reported a strong synergistic effect of ascorbic acid on the action of levofloxacin. The discrepancy between the studies may result from the different concentrations of ascorbic acid used in the tests.

Despite the lack of in vitro synergism, we cannot rule out the positive effect of the ascorbic acid supplementation during fluoroquinolone therapy. However, there are no such studies. There are meta-analyses confirming the positive impact of ascorbic acid supplementation in people with respiratory tract infections [64, 65]. Research on the influence of ascorbic acid supplementation in patients with pneumonia or cystic fibrosis is also ongoing. However, the latest meta-analyses agree that there is no strong evidence and insufficient quality of the research [66, 67].

Hospitalized patients who developed a UTI have changed physiology. Effective glomerular filtration and selection of an antibiotic achieving high concentrations in both serum and urine are key for effective therapy. Fluoroquinolones are a therapeutic option in uncomplicated nephritis and cystitis accompanied by kidney stones. Sub-inhibitory concentrations of antibiotics obtained in vivo may lead to the selection of resistant strains. However, fluoroquinolones at sub-inhibitory concentrations have been proven to lower biofilm formation in vitro. Thus, they can efficiently eliminate the biofilm of susceptible strains.

## Conclusions

The results of our research suggest a beneficial impact of ascorbic acid with rutoside supplementation in UTIs prophylaxis. The prophylaxis may lead to reducing antibiotic (fluoroquinolones) usage. This hypothesis needs verification by checking the correlation between the course of UTI (duration, severity of symptoms, frequency of relapses) and supplementation with rutoside and vitamin C in patients treated with and without fluoroquinolones.

## Data Availability

Not applicable.
